# Topics of Public Interest

**Published:** 1918-07

**Authors:** 


### TOPICS OF PUBLIC INTEREST

**Medical Dept. U. S. Army.** The (regular) Medical Corps (May 10) consisted of 869 officers, 1 major general, 65 colonels, 110 lt. Cols., 298 majors and 395 lts. All remaining members of this corps as it was constituted before the war—with possibly minor exceptions—are now of at least the rank of major, there being no captains at all and the lts. being recent appointees. The Medical Corps of the N. A. has been used mainly to furnish commissions for those content with appointments for the duration of the war, and who, for one reason or another could not receive sufficient recognition otherwise; at least this is the logical deduction from its make-up of 3 brigadier generals, 12 colonels, 92 lt. cols. and 8 majors. The M. R. C. consists of 1281 majors, 1205 active), 4629 captains (4277 active), 12,923 lts. (11,208 active, total 18,833 (active 16,690 active).

**Control of Venereal Disease in N. Y. State.** The Whitby bill went into effect May 5. This law resembles in a general way that of Australia and is the most radical in the U. S. It provides for the arrest and detention of those affected and places the inforcement with local health boards. The general principle of release on recognizance or parole is included but if health authorities are not convinced that treatment will be carried out, the patient may be sent to a hospital. Free diagnostic and therapeutic aid will be given as necessary, by the State Dept. of Health. This is a form of cooperation by the state with the work instituted by the Army.

**Women Physicians,** to the number of 1875 have volunteered for military service if needed and a few have been accepted. The Council of National Defense state that more than third of all women physicians have formally listed themselves.

**Aviation Deaths** in this country, since the beginning of the war total 99, beside 3 at Camp Borden, Can.

**The Buffalo Bureau of Water** publishes the following statistics and estimates: (round numbers given) 1913, 50,000,000,000 gallons pumped; successive years: 52, 53, 58, billions and for 1917, 61,470,593,540. Average cost per gallons, $5.93. Average per capita distribution, 339 gallons, divided into industrial use, 100, legitimate domestic use 50, municipal use (street cleaning, fires, etc.) 25, unnecessary waste 164. We have always understood that Buffalo had the most liberal water supply of the world. Chicago pumps 226 gallons per capita, Detroit 189, Cleveland 118, Milwaukee 110. Army cantonments with an adult male population, a disproportionate number of animals and of peak loads, due to regular habits, require about 50 gallons, though a larger supply is regarded by many as desirable.

---

**The Will** of Mrs. Amory Houghton, Jr., leaves $5,000 to the Corning Hospital.

---

**The War Department** authorizes the following statement: Prisoners of war are entitled to receive and send letters, money orders, and valuables, as well as parcels by post (not exceeding 11 pounds in weight), when intended for international mail, free from all postal duties.

Domestic mail and money orders are subject to the regular postage charges and money order fees.

All mail should be plainly addressed to the prisoner of war, giving his rank, full name, and the name of the prison camp where held (if known), followed by ''Prisoner of War Mail, via New York.'' In addition, all prisoner-of-war mail should bear the name and address of the sender written in the upper left hand corner, and in the case of parcel-post packages, the relationship of the sender to the prisoner of war addressed should be clearly stated, immediately following the sender's name. Parcel-post packages for prisoners in enemy countries may not be sent by organizations or societies, and only one such package per month may be sent to any one prisoner of war.

---

**Soldiers and Sailors** of the U. S. forces, when furloughed and traveling at their own expense, will be granted a rate of approximately one cent per mile. This fare will be available on delivery to ticket agent of certificates signed by commanding officers.

---

**Twenty Uniforms** for women in war work are now officially recognized in the U. S. The women wearing them are munition workers, telephone and radio operators, yeomen, employees of the Shipping Board and the Food Administration, Red Cross workers, and the Young Women's Christian Association workers abroad, Woman's Motor Corps of New York, Girl Scouts, and students in the National Service School of the Woman's Naval Service, Washington, D. C.

---

**250 Telephone Girls** have been chosen to go to France as Signal Corps Unit. In addition to conforming to the qualifications set by the U. S. Signal Corps for membership, the operators were required to pass a psychological examination to determine their motives for wanting to go abroad. They will be stationed in groups of 10 in American bases of supplies and points of embarkation and will not be nearer than 23 miles to the front.

---

**Medical Reserve Corps.** More physicians are needed. Volunteers will probably be accepted immediately and commissions forwarded. The slip inclosed with the commission should be signed and returned without delay. Orders may then be expected in 15 days, unless immediate service is requested or an emergency arises. Initial service is usually at a training camp. This is usually the hardest assignment, because of the necessity of changing so many established habits and because a great deal of training must be accomplished in a minimum of time—the standard period being 3 months but the present emergency often tending to shorten the course. Physicians should realize that, as soon as they have accepted their commissions, they are under military orders and must obey promptly and exactly, without question, and conform to regulations new to their experience and which they must take pains to learn.

The average 1000 men commissioned in the M. R. C. is subject to the following losses before or shortly after becoming available for active duty: 31 for physical disability, inaptitude 13, domestic and community needs 4, deaths 3, resignations 10; total 61. Study this list; it will assure you of two things: that there is little reason to fear that you cannot make good in military service and still less chance to get out of the service for any reason because, after entering, you find that it involves some genuine sacrifice and hard work.

Salaries are $2000, $2400 and $3000 for the respective ranks of 1st lt., captain and major. Foreign service gives an increase of 10% and an allowance in lieu of quarters will now be given to all actually maintaining homes for genuine dependent families, amounting to between 20 and 25%. The necessary initial equipment costs somewhere from $150 to $250 and, for the most part, represents clothing, bedding, etc., which would have to be bought anyway. Not more than $50 can be considered as an ultimate expense. Routine personal expenses and renewal of equipment will cost $50-$100 a month, $75 being a fair average. The relative inexpensiveness of military life in war is mainly due to the necessity of living a simple, industrious life. Young men have a good chance to enter the regular Medical Corps and are eligible to promotion to the rank of captain after a year.

**The St. Paul Medical Journal** has been discontinued, to make way for Minnesota Medicine, the official organ of the state society. We trust that the new journal will maintain the standards of the old.

---

**The Service Flag** of the North Shore Branch of the Chicago Medical Society has recently been dedicated. It bears 100 stars. This may be a suggestion for other medical societies. At any rate, we would like to publish a full list of the Buffalo and Western N. Y. physicians in service and, as the men themselves are too modest and too much hurried on receipt of orders to send in their names and the Surgeon General's office too crowded with more necessary work, such lists which will be of permanent historic interest, should be taken up by committees of county and similar societies.

---

**Death of Last Literal Son of the Revolution** is announced from Omaha, May 8: Owen Moore, born in Vernon, Oneida Co., N. Y. 81 years agô, the son of a revolutionary soldier who enlisted at 18 and served at Valley Forge. Several years ago, we announced the death of the last Son of a Revolutionary Veteran and were corrected by Dr. Palmer of Lockport, whose father fought in the Revolution. Dr. Palmer's letter reminded us of a Revolutionary soldier who had a son born in 1843 or 1844 and, while he died and while few Revolutionary veterans could have been alive and procreating so late, there were undoubtedly a good many sons born of Revolutionary soldiers in the 1830's and it is very doubtful if there are not a considerable number still living.

---

**The National Baby Saving Campaign,** aiming to save 100,000 babies a year but not attempting to describe how it will furnish the direct evidence, has resulted in the appointment of the following local committee for Buffalo, by Dr. Franklin C. Gram, Acting Health Commissioner; Drs. Edward Durney, Carl G. Leo-Wolf, Douglas P. Arnold, H. K. De Groat and Mrs. Anna Hanson.

---

**Birth and Death Registration.** 27 states, the District of Columbia, the territory of Hawaii, and 43 cities in other states are now included in the area for which the Census Bureau publishes death statistics. The registration area started in 1902 with about 40% of the population, remained unchanged till 1906, and now includes about 75% of the population.

Similar collection of birth statistics began two years ago with about 31% of the population—N. Eng., N. Y., Pa.,

Mich., Minn., D. C. The addition of Md., Va., N. C., Ky., O., Ind., Wis., Utah and Wash. brings the part of the population covered up to 51%. The problems of military registration have emphasized the need of extending accurate collection of vital statistics to the whole country.

---

**The Red Cross and Animal Experimentation.** In view of the protests by antivivisectionists, the Red Cross has decided to discontinue its appropriations for animal experimentation. The Buffalo Academy of Medicine has recently filed a counter-protest, as have probably many other medical organizations. The argument in favor of proper use of laboratory animals needs no discussion in a medium of this kind. However, the medical profession should not ignore certain reasons of expediency. The Red Cross is an exceedingly necessary adjunct to the government in the conduct of war, not only by relieving the official channels from the collection of a large amount of money by taxation and of various duties that can in many ways be discharged by the more elastic methods of an unofficial organization better than by government officers, especially when haste, at the disregard of formalities, is required. Red Cross funds and the actual work, depend largely on popular sentiment and on the sentiment of that part of the community which always has looked with disfavor on animal experimentation. Animal experimentation is not a logical function of the Red Cross itself; it can merely assist by turning over part of its funds and it is a very practical question whether an insistance on co-operating with experimental and therapeutic laboratories might not reduce the receipts in dollars and donated material and labor to a still greater degree. The immediate problem is not of scientific logic or ultimate ends but of actual fact. If, as seems probable, ample funds for animal experimentation can be secured more directly, and if the Red Cross can, by cutting out its appropriation, gain for its humanitarian work of a directly practical nature, the emergency is too great to try to force this issue.

---

**Centenarian.** Mrs. Antoinette Smith of Springfield, Ill., celebrated her 106th birthday late in May. She was born in the Madeira Islands, has 5 children living, the oldest aged 80, and other descendants down to a great great grandchild.

---

**Military Mortality.** The total deaths among British army surgeons, from the beginning of the war to Jan. 1, are said to have been only 782. The present medical staff numbers about 14,000, but, of these 1200 are Canadians and there are also—or rather were at that date, a small percentage of Americans, the proportion having been considerably increased recently. A fair basis for computing death rate, is 12,000. The death rate therefore corresponds to scarcely 2% per annum, which is about the normal for physicians of all ages though necessarily greater than for the profession excluding those of advanced years. The total death rate from disease has been about 4%, or a trifle over 1% per annum, as against 67% for the period of the Napoleonic wars. Deaths from military causes are not stated but it is said that 2% of those exposed to gas have died and that shell shock, which American authorities are inclined to consider neurotic, has caused a fraction of a per cent. of deaths.

---

**The Chautauqua Co. Tuberculosis Hospital**, for which Mrs. Elizabeth M. Newton of Fredonia left $150,000, is being erected on the south shore of Cassadaga Lake, work being rushed to accommodate incipient cases among soldiers. It may be remarked that the alarm as to tuberculosis in the army is unfounded. While approximately 10% of the total mortality of any average community has been due to tuberculosis for many years, only about 1% of soldiers are found to be affected with active lesions, the diagnosis being most searching and detecting cases that would be passed over in civilian life. With remarkably few exceptions, the cases are detected early enough to assure a permanent cure if due care is assured. Indeed, the soldiers are retained long enough to give them a rest and abundant nourishment and to train them in personal hygiene. If the home community will take up the work where the Army leaves off, instead of alarm, we should congratulate ourselves on the ultimate life saving value of mobilization under modern conditions.

---

**The National Committee for Mental Hygiene** announced the establishment of a war emergency course at Smith College, Northampton, Mass., to prepare workers to assist in the reclaiming of soldiers suffering from nervous and mental diseases, including war neuroses and the so-called shell shock.

The prevalence of mental and nervous disorders is unprecedented in the present war, and all of the allied nations have had to provide extensive facilities for dealing with these cases. Many of the disorders yield readily to treatment, which requires the use of civilian aides as well as medical specialists to restore the men to mental soundness and to enable them to adjust themselves again to civil or military life.

The course will be conducted by Smith College and by the Boston Psychopathic Hospital under the auspices of The National Committee for Mental Hygiene, through a sub-committee composed of Dr. E. E. Southard of Boston, Chairman; Dr. William L. Russell and Dr. L. Pierce Clark of New York; Dr. Walter E. Fernald of Waverly, Mass., and William A. Neilson, Ph. D., President of Smith College.

The course will cover eight months, and will be open to college graduates and other young women who have had an equivalent technical training. The academic instruction will be given at Smith College from July 8 to August 31, to be followed by six months' practice work to be given at various centers where there are opportunities for social work with psychiatric cases under the direction of trained social workers. The major studies in the course will be sociology including methods of social case work, psychology and social psychiatry. Minor studies will include hygiene, occupational therapy, military usage, and the writing of records and reports.

---

**Refuse to Recognize Medical Sects for Military Purposes.** The Surgeon General has declined to endorse a movement to recognize various medical sects as such though, as a matter of policy and of fact; competent medical men are eligible to appointment in the various medical corps, irrespective of school of graduation and due regard is shown to individual opinions in therapeutics after appointment. It may be remembered that the same problem was put before President Lincoln, in a limited form, and effectually disposed of by one of his epigrams.

---

**Mail** written and mailed by soldiers, sailors and marines assigned to duty in a foreign country, engaged in the present war, is sent postage free.

---

**Blood Groups.** Untoward results are due to agglutination of the donor's blood by the recipient's. The reactions are shown by the following semi-diagrammatic scheme, arrows showing the direction in which blood may be donated. Donation of blood must never be made against the direction of the arrows. (Note that the double headed arrow in the diagonal between 2 and 3 represents mutual danger). Any group may receive blood safely from its own group. If, as in emergencies, transfusion is practiced without examination of groups, 10% (Group 1) would have 100% of safety; 40% (Group 2) would have 83% of safety; 7% (Group 3) would have 50% of safety; 43% would have 43% of safety: Aggregate safety 65.19%.

